# Cloning and sequence analysis of a partial CDS of leptospiral *ligA* gene in pET-32a – *Escherichia coli* DH5α system

**DOI:** 10.14202/vetworld.2018.557-561

**Published:** 2018-04-30

**Authors:** Manju Soman, Mangattuparambil Mini, Siju Joseph, Jobin Thomas, Nirmal Chacko, T. G. Sumithra, R. Ambily, Binu K. Mani, Rinsha Balan

**Affiliations:** 1 Department of Veterinary Microbiology, College of Veterinary and Animal Sciences, Mannuthy, Thrissur - 680 651, Kerala, India; 2 Department of Veterinary Microbiology, College of Veterinary and Animal Sciences, Pookode, Wayanad, Kerala, India; 3 Unidad de Sanidad y Biotecnologia (SaBio), Instituto de Investigación en Recursos Cinegéticos (IREC), Universidad de Castilla-La Mancha (UCLM), Ronda de Toledo s/n 13071, Ciudad Real, Spain; 4 Department of Animal Husbandry, Veterinary Dispensary Balanthode, Panathady, Kasargod,Kerala, India; 5 ICAR-Central Marine Fisheries Research Institute, Post Box No. 1603, Ernakulam North, Cochin - 682 018, Kerala, India

**Keywords:** cloning, *Escherichia coli* DH5α, *Leptospira*, *ligA*, pET-32a, phylogenetic tree

## Abstract

**Aim:**

This study aims at cloning, sequencing, and phylogenetic analysis of a partial CDS of *ligA* gene in pET-32a - *Escherichia coli* DH5α system, with the objective of identifying the conserved nature of the *ligA* gene in the genus *Leptospira*.

**Materials and Methods:**

A partial CDS (nucleotide 1873 to nucleotide 3363) of the *ligA* gene was amplified from genomic DNA of *Leptospira interrogans* serovar Canicola by polymerase chain reaction (PCR). The PCR-amplified DNA was cloned into pET-32a vector and transformed into competent *E. coli* DH5α bacterial cells. The partial *ligA* gene insert was sequenced and the nucleotide sequences obtained were aligned with the published *ligA* gene sequences of other *Leptospira* serovars, using nucleotide BLAST, NCBI. Phylogenetic analysis of the gene sequence was done by maximum likelihood method using Mega 6.06 software.

**Results:**

The PCR could amplify the 1491 nucleotide sequence spanning from nucleotide 1873 to nucleotide 3363 of the *ligA* gene and the partial *ligA* gene could be successfully cloned in *E. coli* DH5α cells. The nucleotide sequence when analyzed for homology with the reported gene sequences of other *Leptospira* serovars was found to have 100% homology to the 1910 bp to 3320 bp sequence of *ligA* gene of *L. interrogans* strain Kito serogroup Canicola. The predicted protein consisted of 470 aminoacids. Phylogenetic analysis revealed that the *ligA* gene was conserved in *L.interrogans* species.

**Conclusion:**

The partial *ligA* gene could be successfully cloned and sequenced from *E. coli* DH5α cells. The sequence showed 100% homology to the published *ligA* gene sequences. The phylogenetic analysis revealed the conserved nature of the *ligA* gene. Further studies on the expression and immunogenicity of the partial LigA protein need to be carried out to determine its competence as a subunit vaccine candidate.

## Introduction

Epidemics of leptospirosis continue to occur in tropical developing countries, due to poor standards of sanitation and hygiene that leave people and animal at risk to the disease [1]. Leptospirosis is caused by a *Spirochaete* of the genus, *Leptospira*, which comprises about 22 genomospecies, further divided into about 300 antigenically different serovars [2,3]. Recently, 12 new species of *Leptospira* have also been identified [4].

Currently available whole-cell inactivated vaccines provide only serovar-specific, short-term immunity and can afford little cross-protection against the different leptospiral serovars. Genus-specific leptospiral proteins that are conserved throughout the different serovars of *Leptospira* which areimmunogenic and uniquely expressed during acute infection may help in the development of an effective vaccine for leptospirosis as well as aid in studies on its pathogenesis [5]. Recombinant DNA technology aids in the production of purified recombinant genus-specific proteins in bulk quantities and hence helps in the development of subunit vaccines for various infections.

This study aims at cloning of a highly immunodominant region of the *ligA* gene of *Leptospira* in the pET32 vector - *E. coli* DH5α system followed by sequencing and phylogenetic analysis of the partial *ligA* gene.

## Materials and Methods

### Ethical approval

All the procedures have been carried out in accordance with the guidelines laid down by the Institutional Ethics Committee and with local laws and regulations

### Amplification of partial CDS of ligA gene

The genomic DNA of *Leptospira interrogans* serovar Canicola was extracted using QIAamp DNA Mini Kit (Qiagen). The extracted DNA was used as template in polymerase chain reaction (PCR) for amplification of partial CDS of *ligA* gene. Oligonucleotide primers (LigA F and LigA R) were designed for a 1491 bp nucleotide fragment, corresponding to nucleotides 1873-3363 of the complete CDS of *ligA* gene of *L. interrogans* serogroup Canicola strain Kito (GenBank accession number EU7002671).

**Table:** 

Primers	Sequence	Size, bp
LigA F	GCATA C CAT GG CGTC CTC TAA TAC GGA TAT	30
LigA R	ATA CTCGAG CGT AAC TGG AGT ATA AGA ACT CT	32

Bulk PCR (100 µL) was performed in 50 µL reaction mixture containing 34 µL nuclease-free water, 5 µl 10 X PCR buffer, 1 µl of 10 mM dNTP mix (200 µM), 2 µl (20 pmol) of each of the forward and reverse primers, 5 µL of suitably diluted DNA template, and 1 µl of Jumpstart *Taq* DNA polymerase. The PCR amplification cycle comprised an initial denaturation cycle at 96°C for 30 s followed by 35 cycles of denaturation (96°C for 15 s), annealing (55°C for 30 s) and extension (68°C for 2 min), followed by a final extension cycle of 68°C for 5 min. The amplified PCR product of 1491 bp was eluted from the agarose gel using GeneJET^™^ Gel extraction kit, Thermo Scientific^™^, to obtain the purified DNA.

### Cloning of partial CDS of ligAgene

The 1491 bp PCR product obtained from *L. interrogans* serovar Canicola and pET-32a DNA was eluted from the agarose gel using GeneJET Gel extraction kit, Thermo Scientific. Gel-purified *ligA* DNA and gel-purified pET-32a DNA were digested with restriction enzymes, *NcoI* and *XhoI* (MBI, Fermentas). The digested products were again gel purified and subjected to ligation reaction using T4 DNA Ligase (MBI Fermentas). The ligation reaction mixture was incubated for 1 h at 22°C and further kept at 4°C overnight. Competent cells of *E. coli* DH5α^™^ were prepared by calcium chloride method as described by Sambrook and Russell [6] with some modifications. The pET-32a *ligA* DNA was transformed into competent *E. coli* DH5α cells by heat shock method. Five microliters of ligation mixture was mixed with 200 µL of *E. coli* DH5α competent cells and kept on ice for 1 h. These cells were exposed to heat shock at 42°C for exactly 90 s and immediately kept on ice for 5 min. About 800 µL of Luria–Bertani (LB) broth with ampicillin (100 mg/mL) was added to the transformed cells and incubated at 37°C for 45 min. The cells were centrifuged at 6000 × *g* for 8 min and the supernatant was discarded retaining 100 µL of media to resuspend the cells. The transformed cells were plated on LB agar plates containing ampicillin. Appropriate negative controls with untransformed *E. coli* DH5α^™^ cells were also processed simultaneously and the plates were incubated at 37°C for 24-36 h. The recombinant clones were screened by colony PCR. The pET-32a *ligA* plasmid construct was extracted from the transformed *E. coli* DH5α using Thermo Scientific Gene JET plasmid Miniprep Kit and subjected to sequencing.

### Sequencing and analysis of the partial CDS of ligA gene

The partial *ligA* gene insert of pET-32a *ligA* plasmid was submitted to DNA sequencing facility at SciGenom Services, Kakkanad, Cochin, for nucleotide sequencing using the SP6 and T7 promoter primers by dideoxy chain-termination method [7]. The nucleotide sequences obtained were aligned with the published *ligA* gene sequences of other *Leptospira* serovars, using nucleotide BLAST, NCBI.

### Phylogenetic analysis

The phylogenetic analysis involved 8 nucleotide sequences. The sequence alignment was done by ClustalW method in the BioEdit software. Evolutionary analyses were conducted in MEGA 6.06 software [8]. The evolutionary history was inferred using the maximum likelihood method based on the Tamura–Nei model. Initial tree(s) for the heuristic search were obtained automatically by applying Neighbor-Join and BioNJ algorithms to a matrix of pairwise distances estimated using the maximum composite likelihood approach, and then selecting the topology with superior log-likelihood value.

## Results

**Figure-1:**
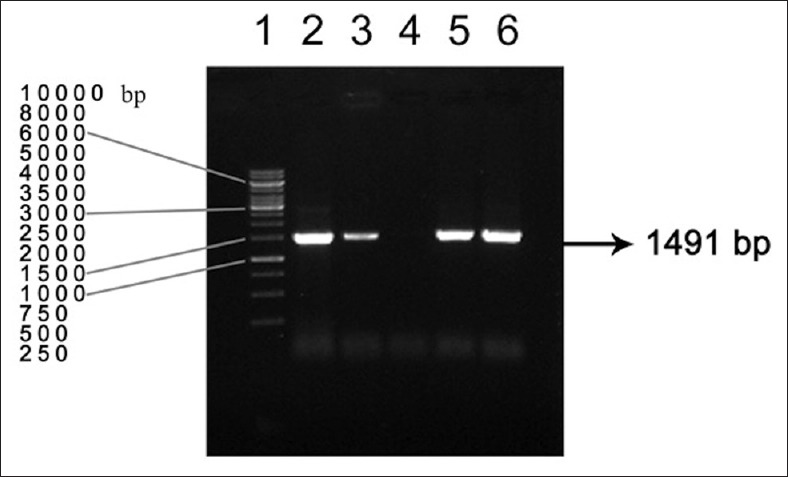
PCR amplification of *ligA* gene Lane 2,3,5,6- positive ampliconsLane 4- negative control Lane 1-DNA marker

The genomic DNA isolated from *L. interrogans* serovar, Canicola, using QIAamp DNA Mini Kit (Qiagen) was of high purity and required concentration (69.1 ng/µL). Primers were designed for the 1491 bp fraction corresponding to nucleotides 1873-3363 of the complete CDS of *ligA* gene, with addition of primer tag regions and RE sites for *NcoI* and *XhoI*. The annealing temperature for the *ligA* primers was determined as 55°C. The PCR could amplify the 1491 nucleotide sequence spanning from nucleotide 1873 to nucleotide 3363 of the *ligA* gene (Figure-1).

**Figure-2:**
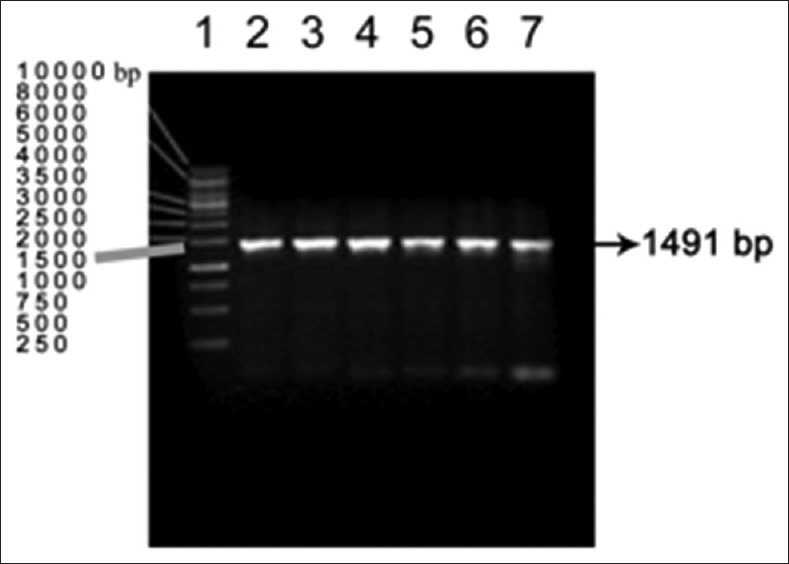
PCR amplification of *E.coli* DH5α clones Lane 2 to 7 –positive amplicons Lane1- DNA marker

The pET-32a-transformed *E. coli* DH5α plated on LB agar with ampicillin yielded 25-30 colonies following 36 h of incubation at 37°C. Control plates inoculated with untransformed *E. coli* DH5α did not yield any colonies. Colony PCR using LigAF and LigAR primers revealed bands at 1491 bp size (Figure-2) on the agarose gel which indicated positive cloning.

### Nucleotide sequencing and analysis of partial CDS of ligA gene

The nucleotide sequence of the partial *ligA* gene insert of the pET-32a *ligA* plasmid construct, when analyzed for homology with the reported gene sequences of other *Leptospira* serovars was found to have 100% homology to the 1910 bp to 3320 bp sequence of *ligA* gene of *L. interrogans* strain Kito serogroup Canicola and 99% homology to *ligA* gene (1910-3320 bp) of *L. interrogans* serovar Kennewicki strain PO-06-047 and *ligA* gene (1910 bp to 3320 bp) of *L. interrogans* serovar Pomona isolate pLPLIGA. The nucleotide sequence has been published in Genbank database of NCBI with the accession number KX964647. The predicted protein, Protein ID APG21200.1, consisted of 470 aminoacids which showed 100% homology to bacterial immunoglobulin-like domain of *L. interrogans* strain L0996, *L. interrogans* serovar Medanensis strain L0448, and *L. interrogans* serovar Canicola strain Fiocruz LV133.

### Phylogenetic analysis

**Figure-3:**
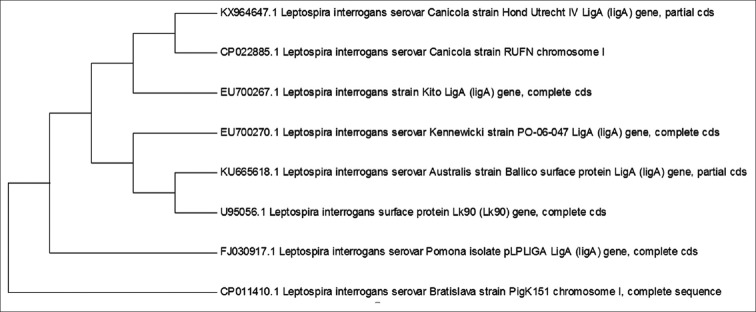
*Leptospira interrogans* serovar Canicola strain Hond Utrecht IV LigA (ligA) gene, partial CDS sequence – phylogenetic tree.

The optimal tree constructed with 8 nucleotide sequences with the highest log likelihood (−3438.4275) is shown in Figure-3.

## Discussion

The Lig (leptospiral Ig-like) protein is a family of surface-exposed lipoproteins found only in pathogenic *Leptospira* and expressed during acute infection and hence is thought to play a role in the pathogenesis of leptospirosis. The LigA, a 130 kDa protein encoded by the *ligA* gene, belongs to the Lig family of proteins comprising of LigA and LigB that possess a series of 90 amino acids tandem repeats homologous to the bacterial immunoglobulin-like (Big) domain. The recombination patterns and sequence variations of *ligA, ligB*, and *ligC* genes, studied in 10 pathogenic strains of five *Leptospira* species, revealed that *ligA* might have been created by partial gene duplication of *ligB* involving two steps. The amino-terminal domains of LigB and LigA proteins, of strains possessing both genes, were found to be identical having 98.5±0.8% mean identity [9]. The LigA proteins are predicted to be lipoproteins as they possess a 17 amino acid N-terminal signal peptide and a lipoprotein signal peptidase cleavage site. The *ligA* gene encoding this protein reportedly comprise of 3675 bp with 12 tandem repeats. It has been reported that cloning of full-length *ligA* gene in pET-22b plasmid and expression in *E. coli* produced very low levels of rLigA, which was attributed to the high toxicity of the protein [10]. Several studies have demonstrated the cross-reactive immunoprotective effect of recombinant *LigAN1* protein, a truncated form of LigA comprising a carboxy-terminal repeat domain unique to the LigA [11-13]. In this study, we have amplified a 1491 bp fraction of the *ligA* gene, within the LigAN1 region, encoding hydrophilic amino acids from 624 to 1121. The amplified DNA sequence was cloned on to pET-32a expression vector using host *E.coli* DH5α. The pET vectors are considered to be one of the most powerful vector systems developed for cloning and expression of bacterial genes in *E. coli* [14]. The pET-32a (+) vector series is designed for expression of peptide sequences fused with the 6X histidine tags. Target genes cloned in pET plasmids are placed under the control of a strong bacteriophage T7 promoter and expression is induced by providing a source of T7 RNA polymerase in the host cell. The T7 RNA polymerase is highly selective and active and helps in targeting almost all of the cells’ resources on the expres­sion of the specific gene. In a study, the parasporin 1 gene of *Bacillus thuringiensis* was amplified by PCR and cloned into pGEM-vector. The predicted protein encoded by the 2371 nucleotides’ long gene sequence was composed of 789 amino acids with an estimated molecular weight of 84 kDa [15]. Vector pRham-SUMO was used to clone and sequence the nagH gene of *C. chauvoei* [16]. In another study, P67 gene of *Mycoplasma leachii* was cloned in pRham N-His SUMO Kan vector and transformed into competent *Escherichia cloni* 10G cells [17]. In this study, the pET-32a *ligA* construct was transformed into *E. coli* DH5α, an expression host that lacks T7 RNA polymerase gene. *E. coli* is the most preferred host in recombinant DNA technology because its genome is well studied, is relatively cheap and has short generation time [18]. The phylogenetic tree revealed that the *ligA* gene of the tested strain of *Leptospira* species occupied the same position in the phylogenetic tree as other reference leptospiral strains of the particular species. This confirmed that the *ligA* gene was conserved in the genus *Leptospira* and hence indicated that the LigA protein was a probable candidate for subunit vaccines. The truncated recombinant LigA protein for vaccine studies may be expressed by subcloning the pET-32a *ligA* construct into *E. coli* BL21 (DE3) expression system and induction with isopropyl β-D-1-thiogalactopyranoside (IPTG) [19].

## Conclusion

The results of the study reveal that the partial *ligA* gene can be cloned into pET-32a-*E. coli* DH5α system. The phylogenetic analysis revealed that the partial *ligA* gene was conserved in the genus *Leptospira*. Hence, further studies need to be conducted on the immunogenicity and vaccine properties of the protein.

## Authors’ Contributions

MS carried out the study. MS prepared the article. MM and SJ formulated the study. JT, TGS, NC, RA, BKM, and RB participated in scientific discussions. All authors read and approved the final manuscript.
